# Atopic dermatitis increases the risk of infective endocarditis in prosthetic heart valve patients – A retrospective study

**DOI:** 10.1016/j.jdin.2024.09.016

**Published:** 2024-11-16

**Authors:** Chapman Wei, Ahmad Mustafa, Radu Grovu, Angela Rosenberg, Alaukika Agarwal, Alanna Davis, Nawal Mustafa, Stefan Bradu, Suzanne El-Sayegh

**Affiliations:** a Department of Medicine at Staten Island University Hospital/Northwell Health, Staten Island, New York; b Department of Dermatology, Barbara Zucker School of Medicine at Hofstra/Northwell, New Hyde Park, New York

**Keywords:** asthma, atopic dermatitis, bacteremia, cannabinoid use, eczema, infective endocarditis, prosthetic heart valve, skin infection

*To the Editor:* Practicing adequate skin hygiene is an important part of infective endocarditis (IE) prophylaxis in prosthetic heart valve patients.^1^ Prosthetic valve patients are at increased risk for contracting IE because of increased bacterial adherence to prosthetic device material. The study of skin conditions like atopic dermatitis (AD) with altered skin microbiota, increased skin inflammation and disrupted skin barrier in this context might identify additional important risk factors that can further increase the IE risk in prosthetic valve patients. We retrospectively evaluated the association of AD with IE in prosthetic valve patients.

Prosthetic valve patients from the National Inpatient Sample Database 2016-2018 were selected. Patients with intravenous drug use, infected central lines, past history of IE, less than 18 years old, and with missing data (age, gender, and race) were excluded.^2^ This cohort was subsequently stratified by AD. Patient demographics and comorbidities were collected (Table I). The primary outcome was IE. Univariate analyses were conducted using chi-square and student’s *t*-tests. Multivariate analysis was conducted using binary logistic regression.

**Table I:** Patient demographics, comorbidities, and infectious endocarditis frequency

Variables	Atopic dermatitis *N* = 353 N (%)	Nonatopic dermatitis *N* = 188262 N (%)	*P* value
Age (y ± SD)	72.17 ± 4.26	72.55 ± 13.86	.605
Female	123 (34.8)	84598 (44.9)	**<.001**
Race			.724
White	150162 (79.8)	287 (81.3)	
Black	17371 (9.2)	32 (9.1)	
Other	20729 (11.0)	34 (10.6)	
Smoking	122 (34.6)	62146 (33.0)	.574
Cancer	23 (6.5)	14482 (7.7)	.466
Prior PPM	49 (13.9)	31099 (16.5)	.207
Prior ICD	23 (6.5)	13751 (7.3)	.641
Dental disease	0 (0.0)	139 (0.1)	>.999
AIDS	2 (0.6)	687 (0.4)	.853
Coronary artery disease	190 (53.8)	102603 (54.5)	.841
COPD	110 (31.2)	49188 (26.1)	**.037**
Diabetes mellitus	117 (33.1)	65836 (35.0)	.507
Dyslipidemia	144 (40.8)	74298 (39.5)	.649
Hypertension	99 (28.0)	62561 (33.2)	**.044**
Peripheral vascular disease	6 (1.7)	4503 (2.4)	.499
Anemia	111 (31.4)	52358 (27.8)	.144
Smoking	122 (34.6)	62146 (33.0)	.574
Atrial fibrillation	139 (39.4)	66478 (35.3)	.123
Chronic liver disease	22 (6.2)	11219 (6.0)	.917
Carotid artery disease	13 (3.7)	4713 (2.5)	.213
Chronic kidney disease	132 (37.4)	63716 (33.8)	.176
Coagulable disorders	53 (15.0)	23138 (12.3)	.140
Chronic heart failure	168 (47.6)	84372 (44.8)	.320
Pulmonary hypertension	49 (13.9)	24393 (13.0)	.662
Prior TIA/CVA	48 (13.6)	27879 (14.8)	.572
Obesity	63 (17.8)	26848 (14.3)	.065
Infective endocarditis	12 (3.4)	3278 (1.7)	**.017**

Bold values indicate a *P*-value <.05.

*AIDS*, Acquired immunodeficiency syndrome; *COPD*, chronic obstructive pulmonary disease; *CVA*, cerebrovascular accident; *ICD*, implantable cardiac defibrillator; *PPM*, permanent pacemaker; *SD*, standard deviation; *TIA*, transient ischemic attack.

Of 188,615 patients, 353 patients had AD and 188,262 patients did not. AD cohort had more males and less rates of hypertension (Table I). AD patients had increased IE rates compared to non-AD patients (1.7%; Table I). After logistic regression analysis, AD had increased risk for IE (adjusted OR: 2.0 [1.1-3.5]; *P* = .023; Table II).

**Table II:** Multivariate and postmatch logistic regression analysis

Variables	Odds ratio	95% confidence interval	*P* value
Multivariate logistic regression			
Atopic dermatitis (adjusted)	2.0	1.1-3.5	**.023**

Multivariate analysis and propensity match were performed using all patient variables. Bold values indicate a *P*-value <.05.

Studies have reported possible links between AD and IE, usually in the setting of active skin infection.^3^^,^^4^ In a retrospective cohort study on 120 patients, 6.7% of IE patients had AD.^3^ Chronic skin diseases, like AD, have been suggested to increase the risk for IE and bacteremia.^1^ This might be due to a change in the skin microbiota and increased skin inflammation with disrupted skin barrier in patients with AD. In most of these cases, the most common pathogen was *Staphylococcus aureus*, which is also the most common for prosthetic valve IE.^5^ The weakened skin barrier can lead to increased bacterial and fungal entry, hence leading to increased susceptibility to fungemia and bacteremia. Thus, optimized treatment of AD with strict cutaneous hygiene, including advice on tattoos and skin piercings, are likely to be important for IE prevention in prosthetic valve patients.^1^

The main limitations of our study are that it is retrospective in nature and uses administrative billing data with International Classification of Diseases, Tenth Revision codes which are prone to error. The main strengths of our study include assessing data from many patients with prosthetic valves at a national level.

In conclusion, AD was independently associated with increased IE in patients with prosthetic valves. Although IE incidence was small, patients with prosthetic heart valves should be advised that it is important to properly optimize the treatment and management of their AD.

## Conflicts of interest

None disclosed.
